# A Study on the Nature of Association between *Demodex* Mites and Bacteria Involved in Skin and Meibomian Gland Lesions of Demodectic Mange in Cattle

**DOI:** 10.1155/2014/413719

**Published:** 2014-08-06

**Authors:** Mukhtar Taha Abu-Samra, Yassir Adam Shuaib

**Affiliations:** ^1^Department of Veterinary Medicine and Surgery, College of Veterinary Medicine (CVM), Sudan University of Science and Technology (SUST), P.O. Box 204, Hilat Kuku, Khartoum North, Sudan; ^2^Department of Preventive Veterinary Medicine, College of Veterinary Medicine (CVM), Sudan University of Science and Technology (SUST), P.O. Box 204, Hilat Kuku, Khartoum North, Sudan; ^3^Research Center Borstel, Parkallee 18, 23845 Borstel, Germany

## Abstract

The nature of association between *Demodex* mites and bacteria involved in bovine demodectic mange lesions and the normal flora inhabiting the skin of noninfected animals was investigated. *Demodex bovis* and *D. ghanensis* mites were isolated from the infected purulent material extracted from skin and meibomian gland lesions, respectively. The mites could not be demonstrated in skin brushings or impression smears from the eyes of noninfected cattle. Pathogenic bacteria (*Staphylococcus aureus* and *Streptococcus pyogenes* (Group A)) and opportunistic organisms (*Proteus vulgaris*, *Pseudomonas aeruginosa*, *Staphylococcus epidermidis*, and *Trueperella pyogenes*) were isolated from skin lesions of demodectic mange, and *Moraxella bovis* and *Staphylococcus aureus* were isolated from meibomian gland lesions. *Bacillus subtilis*, *Escherichia coli*, *Proteus vulgaris*, *Staphylococcus aureus*, *Staphylococcus epidermidis*, and *Streptococcus pyogenes* (Group A) were isolated from skin brushings from noninfected cattle. The nature of association between *Demodex* mites and bacteria in demodectic mange lesions is synergistic and of equal significance. Pathogenic and opportunistic bacteria facilitated the establishment of *Demodex* mites in the lesions produced and provided an excellent microclimate for the mites to propagate and reproduce, resulting in severe and progressive disease. The “high-turnover” granulomatous reaction which characterized the histopathological changes proved that *Demodex* mites and associated bacteria were persistent and immunogenic.

## 1. Introduction

Demodex belongs to a very specialized group of mites which live in the hair follicles and sebaceous glands of various mammals and man, causing demodectic or follicular mange [1–5]. Demodectic mange in cattle is caused by* Demodex bovis* (Stiles 1892) [1, 4]. Transmission usually occurs by direct contact from the dam to her offspring during nursing in the neonatal period and never between host animals of different species [6, 7].

The disease is characterized by the formation of papules, nodules, pustules, and cysts of varying sizes [3, 5, 8, 9]. The predilection sites of the lesions seemed to be the neck, withers, shoulders, and forequarters [3, 5, 9–11]. As the disease progressed, the lesions spread from their original sites to the rest of the body, and in severe infections, most of the skin became involved [7–9, 12]. Many cattle with demodectic mange might have no visible cutaneous lesions and the disease might pass unnoticed. In such cases, the lesions could only be detected by running the hand over the shoulders, axillae, brisket, and neck and by rolling the loose skin in the axillae and brisket between the thumb and other fingers [3, 5, 9, 13]. A satisfactory diagnosis of demodicosis could only be made by the demonstration of* Demodex *mites in the infected purulent material extracted from nodules and pustules [2, 5, 8, 9, 14].

Meibomian gland demodicosis was reported by a few workers [15–17]. Meibomian glands and eye infection with demodectic mange in cattle were associated with skin lesions of the disease and were never observed in cattle without skin lesions [16]. Some workers [15, 18–20] reported the occurrence of different species of* Demodex *mites in macerated or histological sections of the eyelids of clinically healthy cattle, while other workers [16, 17, 21] described a bilateral palpebral demodicosis with firm swellings in both eyelids. They added that the eyelids became thickened and resulted in blindness due to their physical closure.

The bacteria associated with the mites in demodectic mange lesions were regarded by some workers as secondary invaders [7, 9]. Some workers [22, 23] reported that the bacteria were introduced in the follicles by being carried on the exoskeleton or in the gut of the mite.

The invasion of a host by pathogenic bacteria may be aided by the production of bacterial extracellular substances (invasins) which act against the host by breaking down primary or secondary defenses of the body [24, 25]. Spreading factors are bacterial enzymes that affect the physical properties of tissue matrices and intercellular spaces, thereby promoting the spread of the pathogen [25].

*Staphylococcus aureus *and* Streptococcus pyogenes* were reported to produce a wide array of virulence factors in the form of exotoxins and enzymes that damaged host tissues; expressed many potential virulence factors such as surface proteins that promote colonization of host tissues; inhibit phagocytosis; provoke symptoms of disease; and possessed inherent and acquired resistance to antimicrobial agents [24–26].

*Moraxella bovis* caused infectious bovine keratoconjunctivitis, a devastating ocular disease of cattle which occurs worldwide [27]. The organism is an opportunistic pathogen whose virulence is influenced by both host and environmental factors. The virulence of* M. bovis* was attributed to fimbriae, which allowed adherence of the organisms to the cornea, and during replication, haemolysin and other lytic enzymes were produced playing a significant role in virulence [9, 24, 27–29].

*Pseudomonas aeruginosa* caused a wide range of opportunistic infections. Pathogenic strains of* P. aeruginosa* produced a variety of toxins and enzymes which promote tissue invasion and damage [24, 25]. Attachment to host cells is mediated by fimbriae. Colonization and replication are aided by antiphagocytic properties of exoenzyme S, extracellular slime, and outer membrane lipopolysaccharides. Resistance to complement-mediated damage and the ability to obtain iron from host tissues are additional virulence factors [24, 25].

*Trueperella pyogenes* (*Arcanobacterium pyogenes*) is one of the most common opportunistic pathogens of domestic ruminants, capable of producing suppurative lesions in any organ or tissue in animals. In farm animals, especially ruminants, it is the most common bacteria found in infected wounds and abscesses [30, 31].* A. pyogenes* expressed several known and putative virulence factors required for adherence, subsequent colonization, and host tissue damage [24, 31].

The pathology of the disease was described in different animals including man; in cattle [11, 32]; in the American bison [13]; in dogs [33, 34]; and in man [35, 36].

Previous workers have undermined the role played by bacteria in demodectic mange lesions by simply regarding them as secondary invaders. The current study is probably the first encounter on the nature of association of bacteria and* Demodex *mites in skin and meibomian gland lesions of demodectic mange. Studying the normal flora inhabiting the skin of normal noninfected cattle of the same herds is a crucial and prerequisite part of this investigation, as it represents an important integral part in the ecology of cattle skin. Moreover, the role played by bacteria in the severity and spread of the lesions of demodectic mange in relation to host parasite interactions is elucidated.

## 2. Materials and Methods

### 2.1. Animals

Three hundred cattle with skin and eye lesions suggestive of demodectic mange and 50 noninfected cattle belonging to the same herds were clinically examined and sampled to study the nature of association between* Demodex *mites and bacteria involved in the lesions of demodectic mange. Purulent infected material was extracted from skin and meibomian gland lesions using sterile techniques. Each specimen of infected material was divided into two parts. The first part of each specimen was kept in a sterile Bijou bottle and refrigerated for bacteriological investigations. The second part was kept in other Bijou bottles containing equal volumes of glycerol and ethanol for parasitological examination. Two sets of impression smears and swabs from the eyes of noninfected cattle were also collected and refrigerated. Bacteriological and parasitological investigations were also conducted on each of 200 specimens of skin brushings collected from noninfected cattle belonging to the same infected herds using a coarse brush. Skin biopsy specimens were collected from the infected and noninfected cattle following the technique described by Abu-Samra [37]. After being slaughtered, the upper and lower eyelids of the eyes from 25 cattle with eye infection and 10 noninfected ones were excised and removed. The biopsy and necropsy specimens were fixed in 10% formal saline.

### 2.2. Laboratory Investigations

#### 2.2.1. Bacteriological Investigations

Two milliliters of sterile nutrient broth was added to the refrigerated infected material and skin brushings in each bottle, and two drops of sterile nutrient broth were placed on the swabs from the eyes of noninfected animals. The contents of the bottles were thoroughly mixed using a mechanical shaker. The procedures adopted for the preparation of culture media and media for biochemical tests were according to standard methods and techniques described by Barrow and Feltham [38]. Each of the specimens was cultured under aerobic, anaerobic, and increased carbon dioxide conditions at 37°C for 24–48 hours on the following media: nutrient agar [39], 5 percent sheep, bovine or horse blood enriched agar prepared from blood agar, McConkey's agar, and nutrient broth (Oxoid). Moreover, one set of the seeded blood enriched agar was incubated at 33°C in a humid chamber. Pure cultures were obtained through serial subcultures. The pure isolates were biochemically tested according to standard methods and techniques [38].

#### 2.2.2. Parasitological Investigations

A small piece from each specimen of the infected purulent material was crushed between two microscope slides. Another piece from each specimen and a small amount of each specimen of skin brushings was placed in the middle of a microscope slide, a drop of 20 percent potassium hydroxide was added, and the preparations were gently heated and covered with coverslips. The two preparations were examined under the microscope. Individual mites were isolated and identified following the technique described by Abu-Samra et al. [40].

#### 2.2.3. Histopathological Investigations

The biopsy and necropsy specimens were processed, embedded in paraffin wax, and sectioned at 5 *μ*m prior to staining with Haematoxylin and Eosin stain and examined following standard methods and techniques described by Bancroft and Harry [41].

## 3. Results

### 3.1. Animals

**Figure 1:**
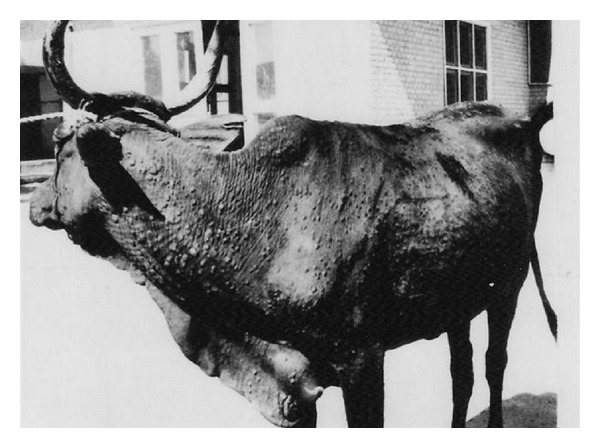
A cow infected with demodectic mange, showing pustules involving the neck, and nodules scattered over the whole body. Note folding of the skin at the base of the neck.

**Figure 2:**
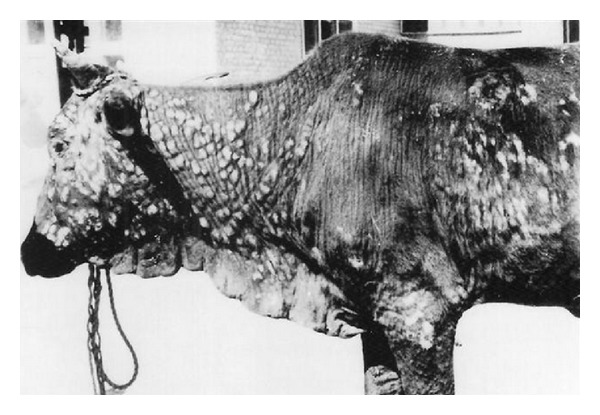
Pustules and crust-covered lesions of demodectic mange involving extensive areas of the body of a heifer. Note marked wrinkling and folding of the skin.

Five forms of skin lesions were recognized. They were papules, nodules and papules, nodules and few pustules (Figure 1), pustules and few nodules or pustules, and crust-covered lesions (Figure 2).  Table 1 summarizes the gross appearance of the five forms of skin lesions.

**Figure 3:**
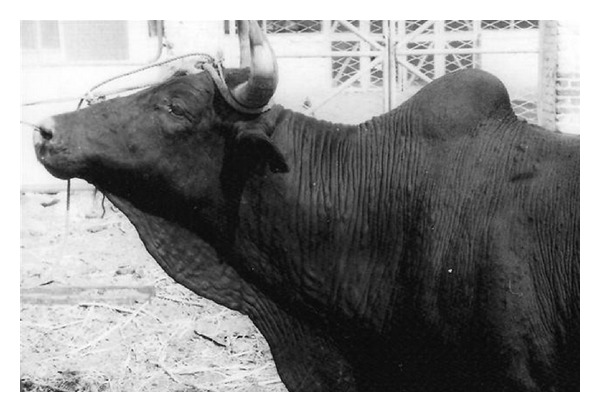
Simultaneous skin and meibomian gland demodicosis. Note swelling of the eyelids and nodules on the upper eyelid arranged in a linear fashion.

Themeibomian gland lesions were characterized by swelling of the eyelids, lacrimation, hyperaemia, and congestion of the mucous membranes, and in extreme cases by purulent exudation, swelling, and closure of the eyelids. Both eyelids showed 2–4 purulent nodules of 3-4 mm in diameter arranged in a linear fashion (Figure 3). Inspection of the eyes was much resented by the animals, and in the majority of animals, the lower eyelids were more affected and disfigured than the upper ones. Among the 300 cattle, 218 (72.7%) animals had simultaneous skin and meibomian gland lesions. The remaining 82 (27.3%) cattle had skin but no eye infection, and their eyes were free of any clinically detectable abnormality. None of the infected cattle had only meibomian gland lesions. All animals had severe pruritus and were persistently scratching, rubbing, licking or gnawing at the affected areas of the skin, and rubbing their eyes against their body.

Control noninfected cattle had no visible or palpable lesions after careful examination of the skin and eyes.

### 3.2. Laboratory Investigations

#### 3.2.1. Bacteriological Findings

*Skin Lesions*. Culture of the 300 specimens of infected purulent material (Table 2) revealed growth of organisms from 252 specimens (84%) and no growth was obtained from the remaining 48 specimens (16%). Gram's stained smears from the cultures revealed the following: Gram-positive cocci in 136 cultures (54%), Gram-negative rods in 106 cultures (42%), and Gram-positive rods in the remaining 10 cultures (4%).

*Meibomian Gland Lesions*. Culture of 218 specimens of infected purulent material (Table 2) revealed growth of organisms from 128 specimens (58.7%) and no growth was obtained from the remaining 90 specimens (41.3%). Gram's stained smears from the cultures showed the following: Gram-negative diplobacilli occurring in pairs in 102 cultures (80%) and mixed Gram-negative diplobacilli and clusters of Gram-positive cocci in the remaining 26 cultures (20%).

*Skin Brushings*. Cultures of 200 skin brushings from noninfected cattle (Table 2) revealed growth of organisms from 156 specimens (78%) and no growth was obtained from cultures of the remaining 44 specimens (22%). Gram's stained smears from the cultures revealed Gram-positive cocci in 75 cultures (48%), Gram-positive rods in 57 cultures (36%), and Gram-negative rods in 24 cultures (16%).

*Eye Impression Smears and Swabs*. Gram's stained impression smears from the eyes of noninfected cattle showed insignificant numbers of microorganisms, and no growth was obtained from swab cultures. 

*Identification of the Isolates*. The following bacteria were isolated and identified following the methods and techniques described by Barrow and Feltham [38]:* Bacillus subtilis, Escherichia coli, Moraxella bovis, Proteus vulgaris, Pseudomonas aeruginosa, Staphylococcus aureus, Staphylococcus epidermidis, Streptococcus pyogenes *(Group A), and* Trueperella* (*Arcanobacterium*)* pyogenes* (Table 2).

#### 3.2.2. Parasitological Findings

**Figure 4:**
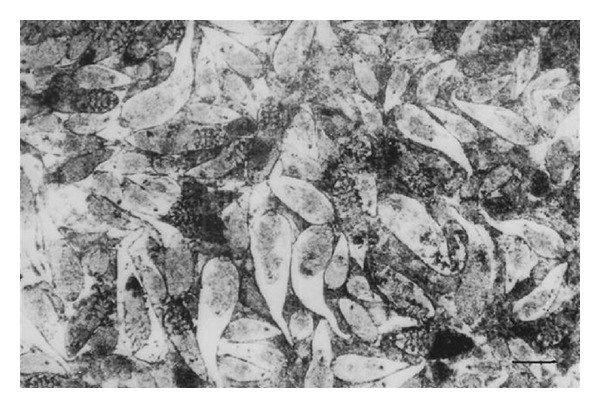
Numerous* Demodex bovis *mites and its different developmental stages, pus, and cell debris in a crushed specimen of infected purulent material extracted from skin lesions of demodectic mange. Scale bar: 70 *μ*m.

*Skin Lesions*. Examination of crushed infected material showed numerous different developmental stages of* Demodex *mites, pus, and cell debris (Figure 4). Eggs, larvae, nymphs, and adult mites were seen in 20 percent potassium hydroxide preparations. The mites were isolated and identified as* Demodex bovis*. They are elongated, gently tapered, and cigar-shaped. 

**Figure 5:**
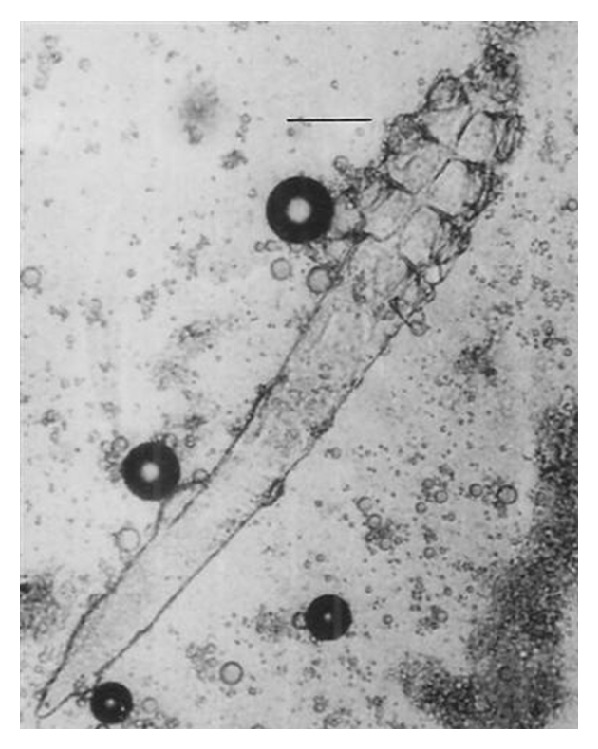
*Demodex ghanensis *mite in infected material extracted from meibomian gland lesions of demodectic mange in a cow. 20% potassium hydroxide solution. Scale bar: 50 *μ*m.

*Meibomian Gland Lesions.* Findings similar to those recorded for skin lesions were observed. However,* Demodex *mites and their different developmental stages were much less than those observed in infected material from skin lesions. The mites were identified as* Demodex ghanensis* (Figure 5). They are long, slender, and gradually tapered to a sharp pointed terminus. 

*Skin Brushings*. Examination of the 200 specimens of skin brushings from noninfected cattle, in 20 percent potassium hydroxide, was negative for* Demodex mites*. 

*Eye Impression Smears*. Examination of the 50 eye impression smears from noninfected cattle in 20 percent potassium hydroxide was negative for* Demodex *mites.

#### 3.2.3. Histopathological Findings

**Figure 6:**
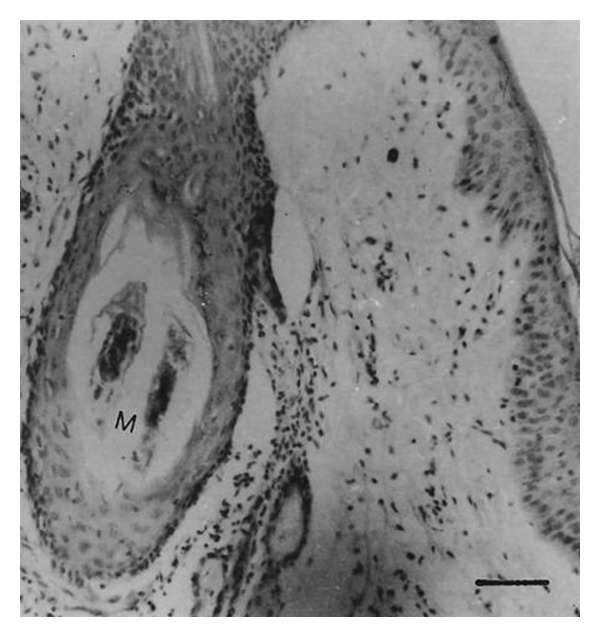
Section from the skin of a cow, showing invasion of a hair follicle with* Demodex bovis*. Note dilatation of the hair follicle bulb, mites (M), and slight infiltration by inflammatory cells in close proximity of the hair follicle. Haematoxylin and Eosin. Scale bar: 100 *μ*m

**Figure 7:**
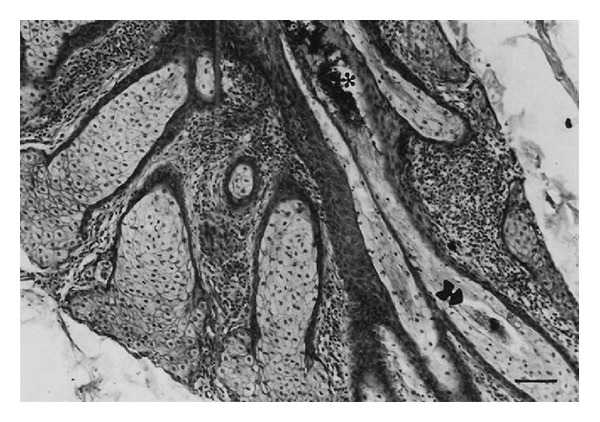
Sections from the eyelid of a cow, showing invasion of the meibomian gland with* Demodex ghanensis*. Note hemorrhage, exudation, and damage of the proximal part of the main collecting tubule (asterisks), marked infiltration with inflammatory cells, dilatation of the main collecting tubules, and* Demodex ghanensis *mite in the main collecting tubule (black arrow). Haematoxylin and Eosin. Scale bar: 120 *μ*m.

**Figure 8:**
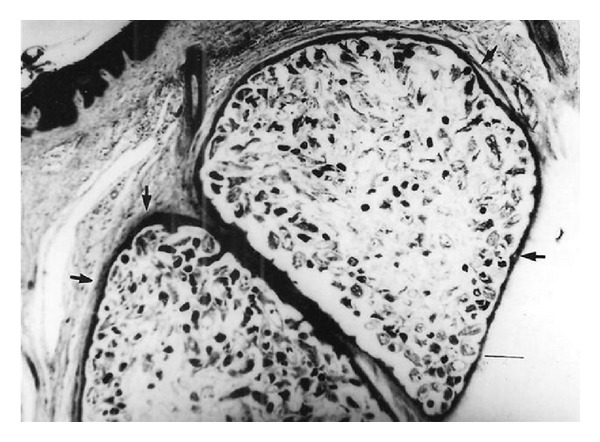
Saccular distension of adjacent hair follicles with* Demodex bovis *mites and associated bacteria, forming large colonies of demodectic mange (bladder-like cysts) in skin sections from an infected cow. Note extremely stretched and jagged epithelial lining (black arrows). Haematoxylin and Eosin. Scale bar: 200**** *μ*m.

**Figure 9:**
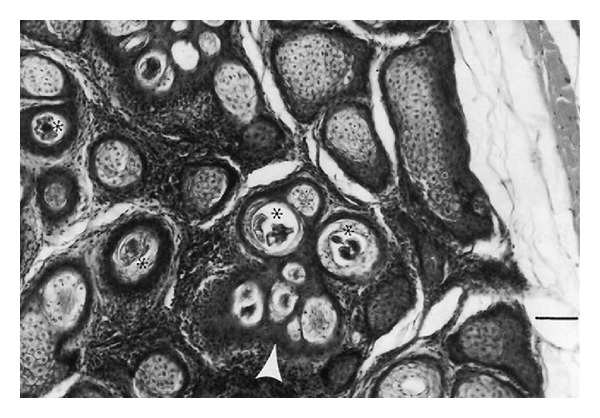
Section from an infected meibomian gland, showing* D. ghanensis *mites (asterisks) in the glandular acini, granulomatous reactions in the infected acini, and proliferation of connective tissue surrounding the granulomas (white arrow head). Haematoxylin and Eosin. Scale bar: 200**** *μ*m.

*Demodex bovis *mites invaded the corium through the orifices of the hair follicles (Figure 6) and* D. ghanensis *invaded the meibomian glands through the orifices of the main collecting tubules (Figure 7). The mechanical movement of the mites through the hair follicles and meibomian glands caused severe irritation, the persistent cutting and feeding of the mite on the epithelium of hair follicles and main collecting tubules of the meibomian glands and their secretions, excretions, and somatic debris resulted in inflammation and dilatation of the orifices of the hair follicles and main collecting tubules of the meibomian glands. This paved the way for active or passive introduction of pathogenic bacteria in the hair follicles and meibomian glands (*Staphylococcus aureus*,* Moraxella bovis, *and* Streptococcus pyogenes* Group A). These pathogenic organisms produced an array of invasins (toxins and enzymes) that break down primary and secondary defenses and produced allergic reactions causing severe irritation and pruritus resulting in scratching, rubbing, licking, or gnawing at the affected areas of the skin and eyes. This produced more inflammation, wounds, and damage of the affected areas in the skin and meibomian glands. The reaction became more severe as the result of invasion of the devitalized structures in the skin and meibomian glands by commensal and opportunistic pathogens (*Proteus vulgaris, Pseudomonas aeruginosa,* and* Trueperella pyogenes*). These organisms also possessed an array of virulence factors, invaded damaged tissues, and produced a suppurative reaction. Thus, the bacteria had created a suitable microclimate for the establishment and rapid replication of* Demodex bovis *and* D. ghanensis* mites in the skin and meibomian gland lesions, respectively. Maximum distension of the hair follicles with mites, bacteria, pus, secretions, and excretions resulted in the transformation of the hair follicles to cylindrical or saccular bladder-like cysts (Figure 8) and dilatation of the main collecting tubules of the meibomian glands (Figure 7). This is exacerbated by the toxins and enzymes produced by the bacteria, and the continuous cutting and feeding of the epithelium of the distended hair follicles and main collecting tubules of the meibomian glands by the mites resulted in damage of these structures and liberation of the mites and primary pathogenic bacteria in the subepidermal and upper dermal layers of the skin and the glandular acini and surrounding connective tissue of the meibomian glands (Figure 9). This resulted in hemorrhage, infiltration by inflammatory cells, and evolved “high-turnover” granulomas with influx of macrophages and lymphocytes. Typical granulomas were seen in areas where mites, bacteria, and purulent exudate congregated (Figure 9). The damaged hair follicles and meibomian gland acini were surrounded by connective tissue, giant and epithelioid cells in the inner layers, and macrophages, lymphocytes, plasma cells, and few eosinophils in the outer layers. The degenerated mites and associated bacteria were engulfed and digested by the giant cells, resulting in regression and later healing of the lesions as judged by the progressive proliferation of connective tissue and degeneration of the granulomatous reaction in different areas of the same section or in different sections.

Sections from the skin and eyelids of noninfected cattle were normal and showed no histopathological changes.

## 4. Discussion

*Demodex bovis *and* D. ghanensis *mites had initiated infection by invading the skin through the orifices of the hair follicles and meibomian glands through the main collecting tubules of the meibomian glands, respectively. They caused inflammation that resulted in dilatation of the orifices of these structures and paved the way for active and/or passive introduction of primary pathogenic bacteria and opportunistic pathogens in the skin and meibomian glands, producing their deleterious damaging effects.

*Bacillus subtilis* and* Escherichia coli *were only isolated from skin brushings from noninfected cattle and seemed to have no role to play in demodectic mange lesions. The former organism is nonpathogenic and is naturally foundin soil and vegetation, and the latter existed in the animals' surroundings as it is commonly found in the lower intestine of warm-blooded organisms [24, 25].

*Proteus vulgaris*,* Staphylococcus aureus*,* Staphylococcus epidermidis*, and* Streptococcus pyogenes *(Group A) were isolated from the infected purulent material extracted from skin lesions of infected cattle and from skin brushings from noninfected cattle. These bacteria had probably chosen the skin surface as a natural habitat, were intestinal flora existing in the animals' surroundings as reported by some workers [9, 24, 25], and/or originated from bladder cysts of demodectic mange which opened towards the exterior liberating their contents on the skin surface of infected animals and resulted in spread of infection as well as contaminating the surroundings, as was reported by other workers [7, 9, 11, 42, 43]. After being actively or passively introduced in the inflamed and dilated hair follicles, pathogenic bacteria produced an array of invasins (toxins and enzymes) that broke down primary and secondary defenses of the body and produced allergic reactions causing severe irritation and pruritus resulting in scratching, rubbing, licking, or gnawing at the affected areas of the skin and evoked severe inflammation, wounds, and damage of the affected areas. These findings confirmed the reports of some workers [25, 26] who enumerated the toxins and enzymes (virulence factors) produced by these organisms which acted against the host by breaking down primary or secondary defenses of the body, aggravating the lesions, and caused marked deterioration of the skin.

*Pseudomonas aeruginosa* and* Trueperella* (*Arcanobacterium*)* pyogenes* were only isolated from skin lesions of demodectic mange and were not isolated from skin brushings of noninfected cattle. The former organism usually infected damaged tissues or tissues with reduced immunity, while the latter is one of the most common opportunistic pathogens of domestic ruminants capable of producing suppurative lesions in any organ or tissue in farm animals. Many workers [25, 30, 31] reported that these organisms produced a suppurative reaction and possessed multiple virulence factors that were instrumental in producing serious damage resulting in marked deterioration of tissues. These organisms caused more damage of the skin lesions and resulted in maximum distension and rupture of the hair follicles resulting in partial or complete liberation of their contents in the surrounding connective tissue resulting in severe pathological changes and evolved “high-turnover” granulomas with influx of macrophages and lymphocytes.

*Moraxella bovis *and* Staphylococcus aureus *were isolated from the infected material extracted from meibomian gland lesions. However, these organisms could not be demonstrated in impression smears or isolated in swab cultures from the eyes of noninfected cattle. This proved that the two organisms did not exist as natural inhabitants of the eyes of noninfected cattle.* Moraxella bovis* is an opportunistic pathogen whose virulence is influenced by both host and environmental factors.* Moraxella bovis* might have been acquired from the animals' surroundings being contaminated by ocular discharges from cattle infected with infectious keratoconjunctivitis, while* Staphylococcus aureus *might have been acquired from the skin when the animals scratched or rubbed their irritated eyes against their bodies.* Moraxella bovis *was reported to be of high morbidity (80%), reaching epizootic proportions when transmission agents (*Musca autumnalis* flies, dust and long grass contaminated by ocular discharges from infected cattle) became available [9]. The pathogenesis of this bacterium was described by many workers [27–29, 44] who reported that it adhered to the cells via its fimbriae and pili proteins, produced *β*-haemolysin toxins which lysed the corneal epithelial cells, and secreted cytotoxic toxin and pathogenic fibrinolysin, phosphatase, hyaluronidase, and aminopeptidases.

Failure to isolate bacteria from 48 specimens (16%) of purulent material extracted from skin lesions of demodectic mange and from 90 specimens (41.3%) of purulent material extracted from meibomian gland lesions was probably due to the destruction of the bacteria by the degenerative and reparative reaction of the high turn-over granulomatous reaction (humoral and cellular responses). The isolation of the mites (*D. bovis *and* D. ghanensis*) from the same specimens was probably due to the chitinous exoskeleton of these mites making them resilient and resistant and would take a longer time to be destroyed, engulfed, and digested by the macrophages and giant cells.

*Demodex bovis *mites were demonstrated and isolated from all specimens of infected material extracted from skin lesions. However, the mite was not encountered in any of the skin brushings from noninfected animals. This finding proved that* Demodex bovis *mites did not exist as normal inhabitants of the skin of healthy normal cattle and was contrary to the findings of many workers [9, 13, 16, 22, 32, 33, 35, 45–47] who reported the existence of different species of* Demodex *mites in harmony with the host and/or commensals as part of the cutaneous flora on the skin of different species of animals and man. Similarly,* Demodex ghanensis *mites were isolated from all specimens of infected material extracted from meibomian gland lesions but could not be demonstrated in impression smears from the eyes or histological sections from the eyelids of noninfected cattle. This finding also proved that* D. ghanensis *mites did not exist as natural inhabitants of the eyes or eyelids of noninfected cattle and was also contrary to the findings of early investigators [15, 18–20, 48] who reported that the mites were demonstrated in macerated and histological sections of the eyelids of clinically healthy cattle.

The isolation of only* D. bovis *from skin lesions and only* D. ghanensis *from meibomian gland lesions of the same animal was of interest and was subject to speculation. The most probable explanation to this finding was that* Demodex *mites have remarkable adaptation to match their unique environment and thateach species of mite possessed distinct anatomical structures that enabled them to pave their way through their habitat and become well established and reproduced. This finding disagreed with the findings of many workers [32, 48, 49] who isolated* D. ghanensis*,* D. bovis, *and a demodicid shorter than* D. bovis *from the meibomian glands of the same animal (cattle), thus establishing the phenomenon of synhospitality.

In both skin and meibomian gland lesions, the histopathological changes seen were compatible with cell-mediated immunity. This was in agreement with the report of a previous investigator [50] who reported that on the basis of histopathological investigations, an immunological response to the parasite seemed to be implied. In the current study the destruction caused by the mites and associated bacteria resulted in a typical granulomatous reaction. The central core of infection composed of mites, bacteria, and purulent exudate was infiltrated by neutrophils and a few eosinophils and surrounded by lymphocytes, macrophages, epithelioid, giant cells, and proliferation of connective tissue. The giant cells engulfed, destroyed, and digested the mites and bacteria, resulting in healing of the lesions as judged by the progressive proliferation of connective tissue and degeneration of the granulomatous reaction in different areas of the same sections or in different sections. This reaction proved that* Demodex *mites and associated bacteria were both persistent and immunogenic producing severe, progressive, and generalized disease as was observed in natural field cases. These findings were in agreement with other workers [51, 52] who reported that when the inflammatory agent was both persistent and antigenic a “high-turnover” granuloma evolved with influx of macrophages and lymphocytes.

## 5. Conclusion

It was concluded that the nature of association between* Demodex *mites and bacteria in demodectic mange lesions is synergistic and of equal significance. Most of the bacteria involved in the lesions possessed an array of virulence factors (toxins and enzymes) causing severe skin and meibomian gland deterioration and damage facilitating the establishment of* Demodex *mites in the lesions produced and provided an excellent microclimate for the mites to propagate and reproduce, resulting in a severe and progressive disease as observed in natural field cases. Furthermore, the “high-turnover” granulomatous reaction which characterized the histopathological changes proved that* Demodex *mites and associated primary pathogenic bacteria are both persistent and immunogenic.

## Figures and Tables

**Table 1:** Gross appearance of the five forms of skin lesions of demodectic mange in cattle.

Form of lesion	Gross appearance of lesions
Papules	Palpable papules, 1–3 mm in diameter, hard in consistency resembling sand grains, detected after running the hand over the shoulders, axillae, brisket, and neck and by rolling the loose skin in the axillae and brisket between the thumb and other fingers. When incised and squeezed, a small amount of white waxy material was expressed.

Nodules and papules	Visible nodules, 5–10 mm in diameter and 3-4 mm raised above the skin surface, and palpable papules. Nodules are in close association or scattered all over the body. Over some nodules small tufts of hair stood out from the general level of the hair coat. Nodules were firm in consistency and when squeezed a yellowish white material oozed in a single stream.

Nodules and few pustules	Visible nodules and few pustules. Pustules, 15–20 mm in diameter and 2-3 mm raised above the skin surface, majority devoid of hair, erythematous on unpigmented areas, less firm than nodules and when squeezed a yellowish white caseated or moist material tinged with blood oozed out in multiple streams.

Pustules and few nodules	Large indurated pustules and few nodules; pustules, 20–30 mm in diameter and 3-4 mm raised above the skin surface, devoid of hair, erythematous on unpigmented areas, fragile and when squeezed a yellowish white material tinged with blood oozed out in large amounts leaving tiny bleeding holes. The skin became thickened showing many wrinkles and folds.

Pustules and crust-covered lesions	Pustules, 20–40 mm in diameter and 2–4 mm raised above the skin surface, in close association, devoid of hair and covered with thin yellowish white crusts. Crust-covered lesions were typified by extensive patches covered with thin yellowish white or pale yellow crusts incorporated with tufts of matted hair. Crusts could easily be removed leaving tiny but visible holes in the skin. The skin became thickened showing many wrinkles and folds.

**Table 2:** Number of bacterial isolates from skin brushings from noninfected cattle and number of isolates from skin and meibomian gland lesions of demodectic mange.

Bacteria isolated	Number of isolates		
	Noninfected cattle	Infected cattle	
	Skin brushings	Skin lesions	Meibomian gland lesions
*Bacillus subtilis *	57	—	—
*Escherichia coli *	9	—	—
*Moraxella bovis *	—	—	102
*Proteus vulgaris *	15	58	—
*Pseudomonas aeruginosa *	—	48	—
*Staphylococcus aureus *	33	80	26∗
*Staphylococcus epidermidis *	16	34	—
*Streptococcus pyogenes* (Group A)	26	22	—
*Trueperella pyogenes* ∗∗	—	10	—

Total	156	252	128

∗Mixed *Moraxella bovis* and *Staphylococcus aureus*.

∗∗Formerly (*Arcanobacterium pyogenes, Actinomyces pyogenes, and Corynebacterium pyogenes*).
